# Revisiting surfactant protein D: an immune surveillance molecule bridging innate and adaptive immunity

**DOI:** 10.3389/fimmu.2024.1491175

**Published:** 2024-12-17

**Authors:** Azra Shamim, Mughair Abdul Aziz, Faryal Saeed, Rekha Kumari, Ann Mary Joseph, Pretty Ponnachan, Uday Kishore, Khaled Masmoudi

**Affiliations:** ^1^ Department Integrative Agriculture, College of Agriculture and Veterinary Medicine, United Arab Emirates University, Al Ain, United Arab Emirates; ^2^ Department of Zoology, A.N College, Patliputra University, Patna, Bihar, India; ^3^ Department of Veterinary Medicine, College of Agriculture and Veterinary Medicine, United Arab Emirates University, Al Ain, United Arab Emirates; ^4^ Zayed Center for Health Sciences, United Arab Emirates University, Al Ain, United Arab Emirates

**Keywords:** SP-D, innate immunity, lectin, PAMP, allergens, apoptosis, cancer

## Abstract

Surfactant protein D (SP-D) is a C-type lectin that was originally discovered as a lung surfactant associated phospholipid recognising protein. It was originally shown to be of great importance in surfactant turnover and homeostasis in conjunction with another hydrophilic surfactant protein i.e. SP-A. In addition, it was found to agglutinate bacteria in suspension and likely a key defence molecule in the lungs. Since its early days of characterization in 1990s, SP-D has turned out to be a central player in the mucosal immunity as pulmonary as well as extrapulmonary innate immune molecule. The most exciting development has been characterization of its C-type lectin or carbohydrate recognition domain (CRDs) that exists in a homotrimeric form in native as well as recombinant versions. SP-D has a range of strategies to recognise pathogen-associated molecular patterns (PAMPs) and thus act as a soluble PAMP-recognizing receptor (PRR), and subsequent destruction of the pathogens directly, or indirectly via phagocytic cells. SP-D also recognizes a range of allergens, competes out with specific IgE antibodies, and downregulates histamine release by basophils and mast cells. These anti-microbial and anti-allergic properties of SP-D have been validated by *in vivo* murine models of infection and allergy. The SP-D gene deficient mice exhibit remarkable phenotypes where lungs are leaky, showing features of fibrosis and emphysema. One of the seminal discoveries in the field has been the observation that activated eosinophils (and other immune cells) can be induced into apoptotic pathways by SP-D. This raised the possibility that SP-D can be an innate immune surveillance molecule. Studies have revealed the ability of a recombinant fragment of human SP-D containing homotrimeric neck and CRD region to induce apoptosis via intrinsic as well as extrinsic pathways; in addition, it also seems capable of interfering with epithelial-to-mesenchymal transition. These studies have opened up enormous possibilities for setting up pre-clinical and clinical trials.

## 1 Introduction

During the respiratory cycle, it is vital for the alveoli to uphold inflation of alveolar sacs to facilitate efficient gas exchange. This is facilitated by the surface-active functions of surfactants which have the ability to decrease surface tension at the air-liquid interface within the alveolar lumen, thus supporting optimal lung function (1, 2). The lung alveolar surface is constituted by type I and type II alveolar epithelial cells. Type II alveolar epithelial cells (AE2Cs) release a combination of phospholipids and surfactant proteins stored in cytoplasmic lamellar bodies into the alveoli (1, 3). The metabolic cycle of surfactants commences with their secretion into the alveolar lumen and ends upon their clearance from the airspace by AE2Cs or alveolar macrophages (2). During gas exchange, surfactants play a crucial role in stabilizing lung volume by preventing alveolar collapse or fluid flooding and aiding in pathogen clearance (1, 4).

Pulmonary surfactant is primarily composed of lipids, particularly phospholipids such as dipalmitoyl phosphatidylcholine (DPPC), which constitute approximately 90% of the complex (2, 5). The remaining 10% consists of proteins, including hydrophobic surfactant proteins (SP) SP-B and SP-C, as well as hydrophilic SP-A and SP-D. SP-A and SP-D are members of the collectin family, large proteins with specific structural domains, with SP-A being the most abundant protein, comprising approximately 3–5%, and SP-D making up around 0.6% of the total surfactant mass (2, 6).

**Figure 1:**
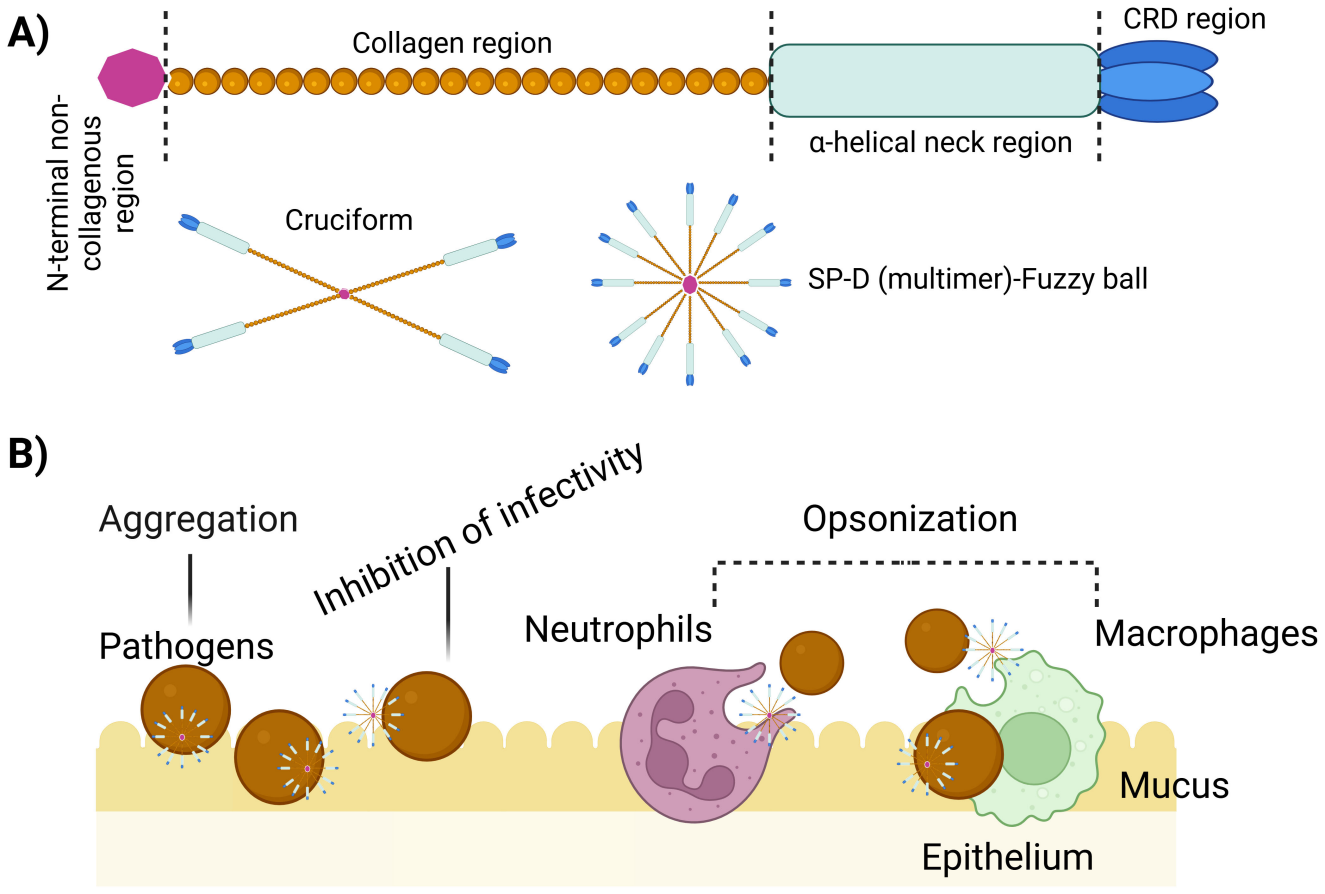
Multimerization and implication of SP-D protein. **(A)** Regions of the structure of a single unit of SP-D protein. These single units can assemble into larger structures such as 4-subunit cruciform or fuzzy ball forms, and structures with more than 4 subunits. **(B)** Illustration emphasizing the role of multimeric SP-D in antimicrobial defence. Multimeric SP-D binds to glycans on microbes, potentially hindering their interaction with receptors, causing microbe aggregation, or acting as an opsonin, thus promoting the uptake of microbes by host cells via endocytosis (10).The killing mechanisms can include superoxidative burst, nitric oxide production and bacteriostatic and/or bactericidal effect.

The introduction of external surfactant containing SP-B and SP-C is a standard practice for treating prematurely born infants who are at risk of developing respiratory distress syndrome (RDS) (1, 7). This surfactant, composed of a blend of lipids, predominantly phospholipids, and proteins including SP-A, SP-B, SP-C, and SP-D, showcases a multifaceted composition (1, 8). These proteins collectively serve diverse functions in surfactant-related activities as well as host defence mechanisms. This review focuses on re-examining the innate immune surveillance molecule SP-D, which plays a crucial role as a link between innate and adaptive immunity. SP-D stands as a sentinel of the immune system, bridging the realms of innate and adaptive immunity with its multifaceted roles in immune surveillance (9, 10). As a member of the collectin (collagen-containing C-type lectin) family, SP-D plays a pivotal role in recognizing and neutralizing a diverse array of pathogens (11–13). Its intricate structure, characterized by its C-terminal carbohydrate recognition domain (CRD), enables SP-D to interact with microbial surfaces, triggering downstream immune responses crucial for facilitating opsonization and subsequent pathogen clearance (13, 14) (**Figure 1**).

SP-D is recognized as a vital element of the innate immune system due to its diverse range of functions and complex interactions with various pathogens and host receptors (13, 15, 16). It exerts regulatory influence over various innate immune cells, including macrophages, neutrophils, and lymphocytes, thereby augmenting their antimicrobial activity. This underscores the potential of SP-D as a therapeutic strategy for pulmonary infections, offering a means to modulate immune homeostasis (17, 18). Moreover, SP-D stands as a pivotal molecule in shaping the adaptive immune response by engaging with antigen-presenting cells such as dendritic cells, and T cells, effectively bridging the gap between innate and adaptive immunity (16, 17; 19–21), and playing a crucial role in host defence mechanisms against a myriad of pathogens, allergens, and cancer, inducing apoptosis in cancer cells and inhibiting epithelial-to-mesenchymal transition (16, 22–24). Furthermore, SP-D levels have been associated with obesity and type 2 diabetes mellitus (25), suggesting its involvement in pulmonary and extra-pulmonary pathologies. SP-D has also been shown as a protective molecule against SARS-CoV-2 infection by binding to spike protein and inhibiting viral entry, replication and interaction with host cells in clinical samples (11, 26).

## 2 Historical perspective

In the mid-20th century, researchers began to investigate the intricacies of pulmonary surfactant, a vital component crucial for respiratory function (1). In 1929, Kurt von Neergaard conducted experiments indicating the presence and importance of pulmonary surfactant in newborns’ first breaths, further highlighting the necessity of a substance within the lungs that could reduce surface tension and prevent alveolar collapse during expiration (27, 28). This revolutionary idea spurred early studies and focused primarily on the lipid components of surfactant, particularly phospholipids, as indispensable for the surface tension-lowering properties of surfactant, marking it a significant milestone in understanding its role in respiratory physiology. Building upon this foundation, almost 25 years later, subsequent research by Richard Pattle, John Clements, and Chris Macklin, studying nerve gas effects on lungs, delved deeper into the composition of pulmonary surfactant, revealing the intricate interplay between surfactant lipids and proteins (1, 28, 29). This collective effort not only elucidated the complex nature of surfactant, but also paved the way for advancement in respiratory medicine and therapeutic interventions aimed at preserving lung function/compliance.

In the 1970s and 1980s, researchers began isolating and characterizing individual surfactant proteins, leading to the identification of several distinct protein components, including SP-A, SP-B, and SP-C. Mary Ellen Avery and Jere Mead’s ground-breaking work further demonstrated that preterm neonates with hyaline membrane disease lacked surfactants, which advanced our understanding by linking surfactant deficiency to respiratory distress syndrome (RDS) in infants, leading to the adoption of surfactant replacement therapy for RDS treatment (28, 30). Initial trials of synthetic surfactant were unsuccessful, but natural surfactants were proven effective in animal models. Tetsuro Fujiwara later demonstrated the efficacy of modified bovine surfactant in preterm infants (28, 31). In the 1980s, randomized trials showed natural surfactants’ superiority over synthetic ones in reducing air leaks and neonatal mortality (28, 32).

Following these advancements, during the 1990s, scientists made additional significant discoveries about surfactant, particularly its role in host immunity against microbial infections and its immunomodulatory effects. It was during these investigations that SP-D was identified as a distinct protein component of innate immunity in the lung surfactant and outside lungs.

### 2.1 Discovery and significance of SP-D

One of the key milestones in the identification of SP-D came in the late 1980s and early 1990s when researchers isolated and characterized a novel surfactant-associated protein from lung lavage samples of various species, including humans and rodents. The discovery and identification of CP-4, now recognized as SP-D, by Persson in 1988, marked a significant milestone in pulmonary research (33, 34). Initially identified as a collagenous glycoprotein through investigations into the composition of pulmonary surfactant secreted by rat type II pneumocytes, CP-4 exhibited unique characteristics that distinguished it from other surfactant proteins, such as SP-A (SP-35) (33). Through meticulous studies involving immune precipitation, chromatography, protease mapping, isolation, purification, and biochemical characterization, researchers confirmed CP-4 as a distinct component of rat pneumocyte culture medium (34). Notably, CP-4 displayed sensitivity to collagenase, contained hydroxyproline, and its secretion was influenced by specific compounds. This detailed biochemical characterization led to the proposal to rename CP-4 as SP-D, aligning with established nomenclature for surfactant proteins (33, 34). Further analyses revealed distinct differences between CP-4 and SP-A in terms of immunological, biochemical, and structural properties.

The discovery of SP-D provided valuable insights into the heterogeneity of collagenous surfactant proteins and highlighted the complexity of the molecular composition of pulmonary surfactant. This finding expanded our understanding of the diverse proteins involved in surfactant function and regulation, shedding light on the intricate mechanisms underlying respiratory physiology.

In 1992, Kuroki’s study confirmed specific binding of SP-D to glucosylceramide (GlcCer), underlining its unique glycolipid recognition property. Calcium-dependent binding to GlcCer and potential interactions with phosphatidylinositol, suggested a crucial role for SP-D in pulmonary homeostatic and defence mechanisms (35). The study indicated that GlcCer, particularly in the form of ceramide monohexoside, served as an endogenous ligand for SP-D, emphasizing its potential involvement in immune responses and defence against microbial invasion in the lungs (35). These findings highlight the significance of SP-D in recognizing specific glycolipids, crucial for pulmonary immune function. The study also anticipated that SP-D might hinder the invasion of bacteria and viruses alongside SP-A through direct binding with microorganisms, or by competing with them for glycolipid receptor binding on cell surfaces (35).

While originally identified in the lungs, subsequent research revealed the presence of SP-D at various mucosal sites in close contact with pathogens. In rats, SP-D was also found in mucus-secreting cells of the gastric mucosa, suggesting a potential role in mucosal defence at the gastric site (36). This broader distribution of SP-D beyond the lungs suggests its involvement in innate immune defence at multiple mucosal surfaces, highlighting its versatility in recognizing and responding to microbial threats. The presence of SP-D in tears and amniotic fluid, and at foetal membranes and gastric mucosa underscores its importance in mucosal defence and immune surveillance against pathogens in various tissues, including during pregnancy (37, 38).

## 3 Structural aspects of surfactant protein D

SP-D is a hydrophilic (15, 39), collagenous glycoprotein and a calcium-dependent member of the collectin subfamily within the C-type lectin superfamily (16, 39). It features a distinctive structure comprising various domains, each intricately contributing to its crucial role and versatilities in innate immune surveillance (24).

SP-D is made up of twelve identical 43 kDa polypeptides with a total molecular weight of ~520 kDa (40) exhibiting a very large (8-9 nm diameter) cruciform-like architecture, composed of 12 subunits arranged in four trimeric units (130kDa), each trimeric unit consisting of three subunits (16) (**Figure 1A**). Within each subunit, four primary domains collectively contribute uniquely to the protein’s ability to recognize and interact with pathogens, modulate immune responses, and facilitate pathogen clearance (16, 41, 42).

The N-terminal domain serves a pivotal role in the protein’s structure and function (6, 43). This cysteine-rich region facilitates oligomerization, promoting the formation of higher-order structures crucial for enhanced ligand affinity and functional activity (6, 16). Additionally, the N-terminal domain contributes to the stabilization of SP-D through disulfide bond-dependent oligomerization, ensuring structural integrity and rigidity (6, 16). N-terminal region itself may not directly participate in ligand binding, its role in maintaining the overall SP-D protein structure is vital for the proper functioning of the CRDs located at the C-terminus of SP-D. The CRDs are responsible for binding to specific ligands on pathogens and other molecules. The CRDs exhibit antimicrobial activity independently of oligomerization, interacting with lipopolysaccharides (LPS) and lipoteichoic acids (6, 44).

The collagenous domain is a triple-helical collagen-like region which is crucial for the protein function. Comprising of Gly-Xaa-Yaa repeats (where X and Y can be any residue following glycine), predominantly with glycine, this domain promotes SP-D multimerization, enhancing its structural integrity (40) (**Figure 1A**). By forming a stable structure, it stabilizes interactions with pathogens and immune cells, enabling effective ligand binding, including to pathogen carbohydrate patterns. Moreover, it activates the immune system through interactions with collectin receptors such Calreticulin/CD91, initiating clearance mechanisms involving pathogens and apoptotic/necrotic cells (15, 45).

The neck region of SP-D is crucial for bridging the N-terminal collagen and C-terminal CRD regions, maintaining the protein’s overall structure and function. This α-helical coiled-coil neck region is essential for trimerizing the CRDs of SP-D, ensuring the stability of its trimeric structure (46, 47). Additionally, the neck region also interacts with phospholipids and acts as a nucleation centre for the trimerisation of CRDs (6, 16).

The homotrimeric C-type lectin or CRD is a critical structural and functional domains found in the SP-D. Positioned at the C-terminal end of SP-D, the CRD serves as a key site for binding to specific carbohydrate moieties such as LPS on bacterial pathogens, allergens, apoptotic, and necrotic cells, and phospholipids. Structurally, the CRD forms a trimeric configuration, facilitated by the α-helical neck region, which is essential for its functional activity. Studies have demonstrated that the trimeric CRDs of SP-D exhibit distinct binding specificities for carbohydrates, LPS, and phospholipids, contributing significantly to pulmonary host defence mechanisms and surfactant reorganization (13, 15, 39, 47).

SP-D utilizes its structural domains to effectively recognize and interact with pathogens, modulate immune responses, and clear harmful entities (**Figure 1B**). A recombinant fragment of homotrimeric CRDs (rfhSP-D) expressed in *Escherichia coli* exhibit stronger ligand affinities compared to monomeric forms lacking the neck peptide (47), highlighting the importance of trimeric organization for ligand interactions.

The oligomeric structure of SP-D enables it to bind a variety of ligands through CRDs on target surfaces, predominantly in a carbohydrate and calcium-dependent manner (48). That allows SP-D to engage in activities such as agglutination/aggregation, of pathogens enhancing phagocytosis, and inhibiting pathogen growth, and viral neutralization (15). Moreover, SP-D regulates inflammation by modulating cytokine production, inhibiting histamine release, and promoting apoptosis in activated immune cells (42). Additionally, the collagen region recruits and activates antigen-presenting cells and T cells for pathogen and apoptotic/necrotic cell opsonization and subsequent phagocytosis, shaping the adaptive immune response (15). Understanding the structure-function relationships of SP-D elucidates how specific structural features facilitate its immune surveillance and defence functions effectively. Its diverse functions include recognizing and neutralizing pathogens, modulating inflammatory responses, and maintaining mucosal homeostasis.

## 4 Multifaceted interactions of SP-D with ligands and protein acceptors

The crystal structures of trimeric CRDs of human SP-D, both with and without ligands, offer valuable insights into the orientation and interaction of bound terminal sugars within the SP-D protein. This structural information is pivotal for understanding the specific binding of SP-D to various ligands such as fatty acids (49, 50), LPS (44), apoptotic cells (51), nucleic acids and lipids including phosphatidylinositol (PI) and glycosylceramides such as glucosylceramide and galactosylceramide. In addition, SP-D also interacts with various protein acceptors, including glycoprotein-340 (Gp-340) and myeloperoxidase (MPO), serving as ligands rather than receptors. The trimeric configuration of SP-D enables multivalent interactions with pathogens, thereby enhancing its binding affinity and efficiency in pathogen recognition (44). Structural and conformational changes occur in the CRDs of SP-D upon binding to various ligands, possibly involving the reorientation of bound terminal sugars (40, 44, 49, 51). These interactions are summarized in **Table 1**, which highlights various ligands that SP-D interacts with.

**Table 1:** Interactions of surfactant protein-D (SP-D) with various ligands.

Interaction/Ligand	Description	References
Fatty acids	SP-D interacts with fatty acids, and the binding is influenced by Conformational changes, calcium and CRDs. Complex, dose-dependent effects observed with Saccharide ligands. physical state of fatty acids affects SP-D’s binding to mannan.	49, 51
LPS (Gram-negative)	SP-D recognizes bacteria via surface mannose, fucose, and N-acetylglucosamine residues in LPS through calcium-dependant interaction. Detailed interaction of the pathogen surface elucidated via crystal structure analysis. Binding to LPS. Binding to LPS shields core structures in SP-D-resistant strains, underscoring the efficiency of recognition and the impact of LPS complexity on binding affinity.	40, 44
Apoptotic cells	SP-D binds to apoptotic cells in a Ca^2+^-independent manner, recognizing various ligands on the cell surface. Triggers events leading to cell clearance and inflammation resolution.	40, 51
Protein acceptors	Interaction with glycoprotein-340 (Gp-340), myeloperoxidase (MPO), C1q (a component of the complement system), immunoglobulins (IgG, IgM, IgE, and secretory IgA), human neutrophil defensins (HNPs), and decorin (a proteoglycan). These interactions are calcium-dependent via CRD and neck domain.	40
MD-2	SP-D binds to MD-2 in a concentration and Ca^2+^ dependent manner. Excess mannose abolishes binding. Co-sedimentation with MD-2 observed in the presence of Ca^2+^.	52
Late apoptotic cells	Strongly binds to late apoptotic cells in a Ca^2+^ independent manner. Its binding behaviour with different cell types and stages of viability involves preferential recognition of various ligands on the apoptotic cell surface, such as nucleic acid, phospholipid, protein, and glycan structures. This binding initiates a cascade of events leading to the recognition and clearance of these cells by immune cells, contributing to inflammation resolution and tissue homeostasis maintenance.	40, 51

SP-D CRDs recognize bacteria, including LPS in Gram-negative bacteria, fungi and viruses by binding mainly to surface mannose, fucose and N-acetylglucosamine residues, and lipids in a calcium-dependent manner (40, 44). The crystal structure of the SP-D-LPS complex elucidated detailed interactions between SP-D and the pathogen surface, highlighting key binding determinants in the SP-D binding pocket (44, 53). SP-D binding to LPS shields core structures in SP-D-resistant strains, underscoring the efficiency of recognition and the impact of LPS complexity on binding affinity.

SP-D collagen domain also contributes to pathogen recognition and binding (15, 16, 43). This domain can interact with other components of the innate immune systems such by binding to receptors on immune cells, initiating downstream signalling pathways, modulating inflammatory responses, enhancing opsonization and clearance of pathogens. Moreover, SP-D can form oligomeric structures like trimers and dodecamers, increasing its binding avidity and enabling multivalent interactions with pathogens. These oligomeric structures enhance its binding affinity to glycosylated ligands on the surface of pathogens, allowing for multivalent interactions and increasing efficiency in pathogen recognition, and further increases its ability to agglutinate pathogens, promote phagocytosis by immune cells, and modulate inflammatory responses. Furthermore, SP-D forms a ring of positive charge in the cleft created by the three CRDs, facilitating interaction with negatively charged ligands on the surface of pathogens.

In addition to the interaction with various lipids, SP-D also interacts via its CRDs with various protein acceptors such as glycoprotein-340 (Gp-340) and myeloperoxidase (MPO); C1q, a component of the complement system; immunoglobulins (Ig), including IgG, IgM, IgE, and secretory IgA; human neutrophil defensins (HNPs) and decorin, a proteoglycan. These interactions occur in a calcium-dependent manner mediated by its trimeric neck and CRD (40) (**Table 2**).

**Table 2:** Cell-surface and soluble receptors of SP-D.

Binding Proteins/Receptors	Domain Localization	Comment	Reference
CD14	SP-D’s Carbon Recognition Domain (CRD) binds to the carbohydrate moiety on CD14, specifically on N-linked oligosaccharides of CD14 receptors present predominantly on monocytes and macrophages. This interaction is Ca^2+^driven, meaning Ca^2+^ is required to facilitate binding between SP-D and CD14.	SP-D binds to both membrane-bound and soluble forms of CD14, a receptor for bacterial lipopolysaccharide (LPS). This interaction competitively inhibits LPS binding to CD14, thereby attenuating LPS-induced inflammatory responses. Consequently, SP-D’s interaction with CD14 reduces the production of pro-inflammatory cytokines such as TNF-α, serving as a crucial regulatory mechanism in modulating lung inflammation.	(10, 54–57)
Calreticulin-CD91 complex	Calreticulin serves as a bridging molecule between SP-D and CD91 (also known as lipoprotein receptor-related protein 1 (LRP1)) on the surface of macrophages and other immune cells, where SP-D binds to calreticulin via its collagen-like domain.	Calreticulin, initially characterized as an intracellular protein, was later discovered to function as part of a cell-surface receptor complex by binding to CD91 (also known as LRP1). This calreticulin-CD91 complex acts as an adaptor or co-receptor, capable of binding the collagenous regions of various proteins including C1q, mannose-binding lectin (MBL), and collectins like SP-D. The interaction between SP-D and the calreticulin-CD91 complex triggers pro-inflammatory responses, enhances phagocytosis of pathogens and apoptotic cells, and modulates cytokine production. Also, this interaction can counteract the local immunosuppression mediated by SP-D’s interaction with SIRPα (Signal Inhibitory Regulatory Protein α).	(6, 40, 58–62)
Signal Inhibitory Regulatory Protein-α (SIRP-α)	SP-D attaches to the membrane-proximal immunoglobulin domain (D3) of SIRPα via a mechanism that depends on both calcium and carbohydrates. This interaction occurs because SP-D recognizes the N-glycosylated residues on SIRPα’s D3 domain. This SP-D-SIRPα interaction is mainly observed in alveolar macrophages.	SP-D binds to SIRPα, it results in an inhibitory signal that prevents activation of mononuclear phagocytes, nuclear factor-κB activation, and secretion of inflammatory cytokines through phospho-p38-dependent signalling. Also, SP-D functions in two ways, either promoting or inhibiting the production of inflammatory mediators based on its binding orientation. When the globular head of SP-D attaches to pathogens, its collagenous tail can engage with calreticulin/CD91, leading to proinflammatory responses.	(6, 10, 14, 58, 63–65)
Human Neutrophil Defensins (HNPs)	HNPs bind directly to SP-D’s carbohydrate recognition domain (CRD).	Human neutrophil defensins (HNPs) inhibit enveloped viruses, by binding specifically to the neck and/or CRD of SP-D. HNP-1, -2, and -3 bind with high affinity, while HNP-4 and human beta-defensins show minimal interaction. HNPs can precipitate SP-D from BAL fluid and reduce its antiviral activity.	(40, 63, 65–67)
Decorin	Decorin, a proteoglycan found in amniotic fluid, interacts with SP-D through a calcium-dependent mechanism. Specifically, SP-D’s CRD binds to the sulfated N-acetyl galactosamine moiety of decorin.	Binding of SP-D to decorin enhances macrophage phagocytosis and facilitates clearance of apoptotic cells and pathogens. The SP-D-decorin complex helps maintain lung tissue integrity and balance immune signals. SP-D also binds late apoptotic cells in a Ca^2+^-independent manner, recognizing various ligands on their surface.	(10, 40, 65, 68, 69)
Myeloperoxidase (MPO)	Intracellular defence molecules present in neutrophils and cell surface during apoptosis	MPO-derived oxidants can modify the structure of SP-D, leading to alterations in its characteristic aggregating activity. Also, MPO serves as a binding molecule for SP-D on late apoptotic neutrophils, where it becomes surface-exposed. This SP-D-MPO is an important mechanism by which neutrophil-derived oxidants can modulate the function of SP-D during inflammation.	(70, 71)
Human osteoclast-associated receptor (OSCAR)	OSCAR binds specifically to the collagenous region of SP-D. OSCAR is found on cell surface of interstitial lung and blood CCR2^+^ inflammatory monocytes.	SP-D binding to OSCAR stimulates TNF-α. This leads to increased inflammation in lungs and other tissues where SP-D accumulates.	(58, 65, 72)
gp-340	Calcium ion dependent protein-protein interaction through Carbohydrate Recognition Domain (CRD). Present on alveolar macrophages.	gp-340 is a soluble SP-D binding protein.	(73–76)
Alveolar Type II Cell Receptors	SP-D interacts with alveolar type II epithelial cells through its calcium-dependent carbohydrate recognition domain (CRD), which recognizes specific carbohydrate structures on these cells, primarily in the alveolar space of the lungs.	SP-D’s interaction with these receptors may influence surfactant homeostasis and immune responses within the alveolar space.	(40, 77, 78)
Myeloid Differentiation factor 2 (MD-2)	Binds in a Calcium dependent manner through the CRD region of SP-D.	The binding of SP-D to MD-2 inhibits MD-2’s ability to facilitate lipopolysaccharide (LPS) binding to TLR4, thereby attenuating the activation of the TLR4/MD-2 signalling pathway. This mechanism helps prevent excessive inflammation caused by LPS.	(40, 79)
Leukocyte Associated Ig-like Receptor-1 (LAIR1)	SP-D interacts with LAIR-1 through its collagenous domain, which binds to LAIR-1, an inhibitory receptor expressed on most immune cells such as neutrophils and monocytes.	The binding of Surfactant Protein D (SP-D) to Leukocyte-Associated Immunoglobulin-Like Receptor 1 (LAIR-1) on neutrophils results in a significant reduction of reactive oxygen species (ROS) signalling. This interaction dampens immune cell activities, including maturation, proliferation, and degranulation of neutrophils showcasing SP-D’s immunomodulatory role.	(65, 80)

Such interactions contribute significantly to the clearance of pathogens and apoptotic cells, as well as the maintenance of pulmonary homeostasis (38, 65). Moreover, SP-D interacts with nucleic acids, specifically DNA and RNA, from different sources, enhancing the uptake of DNA by human monocytes (10). Furthermore, protein acceptors such as Gp-340 play a crucial role in immune scavenging functions. Gp-340 binds SP-D in a calcium-dependent manner via protein-protein rather than a lectin-carbohydrate interaction (81, 82). The immune relevance of this binding is further underscored by its specificity. Although gp-340 does not block the activity of SP-D as a macrophage-stimulating chemotaxis, it can independently promote the random migration of alveolar macrophages (81). This suggests that gp-340 may play a dual role in the regulation of immune responses - by interacting with SP-D and independently activating macrophages (81, 82). Moreover, complex formation between HNPs and SP-D decreases its antiviral activity with a concomitant reduction in total BALF capacity to neutralize virus (40, 65). This interplay demonstrates that the interaction of various immune components within innate immune lineages during respiratory infections is multidimensional. HNP-1 and HNP-2 have also been shown to interact with SP-D in a pH dependent manner, particularly at acidic pH levels (40). However, this binding is specific to SP-D and does not occur with other collectins, which indicate that there are specific mechanisms for the interaction between HNPs and SP-D (40).

SP-D enhances the phagocytic activities of alveolar macrophages, and thus facilitates opsonisation and clearance of apoptotic cells as well as pathogens, which is crucial for lung health by preventing the accumulation of cellular debris and reducing the risk of infection (10, 65, 69). While balancing pro-inflammatory and anti-inflammatory signals to ensure immune homeostasis during respiratory infections or inflammatory challenges the SP-D-decorin complex additionally plays a crucial role in preserving the structural integrity of lung tissue (65, 69). SP-D strongly binds to late apoptotic cells in a Ca^2+^ independent manner unline other collectins such as SP-A which suggests that it utilizes different structural features or binding sites to interact with late apoptotic cells (51, 71). Its binding behavior with different cell types and stages of viability involves preferential recognition of various ligands on the apoptotic cell surface, such as nucleic acid, phospholipid, protein, and glycan structures (40, 51). The binding of SP-D to apoptotic cells triggers a cascade of events leading to the recognition and clearance of these cells by immune cells, contributing to inflammation resolution and tissue homeostasis maintenance.

Additionally, SP-D binds to Myeloid differentiation factor 2 (MD-2) in a concentration and Ca^2+^ dependent manner; excess mannose abolishes this binding. In solution, SP-D co sediments with MD-2 in the presence of Ca^2+^, further emphasizing the role of Ca^2+^ in facilitating this binding (52).

## 5 Putative receptors of SP-D on immune and non-immune cells

SP-D is characterized by versatile binding to multiple binding partners and putative receptors (14, 18, 52, 64, 65, 72, 80).

Recent research has revealed that SP-D modulate cellular functions through two distinct interactions: with signal-inhibitory regulatory protein-α (SIRP-α) and with the CD91–calreticulin complex (14, 64, 65).

SP-D binds to SIRP-α through its CRD region on myeloid lineage cells, where it inhibits the production of pro-inflammatory cytokines like tumor necrosis factor-alpha (TNF-α) and other inflammatory mediators by triggering a specific signaling pathway. This binding triggers the recruitment of SHP-1 and SHP-2, which are protein tyrosine phosphatases that dephosphorylate key signaling molecules involved in pro-inflammatory pathways, including the NF-κB and MAPK pathways, which promotes an anti-inflammatory environment, that is crucial for maintaining immune homeostasis in the lung, effectively suppressing inflammatory responses (14, 64, 65). On the other hand, SP-D interacts with the CD91–calreticulin complex through its collagenous tails, consequently exposing these collagens in an aggregated form on alveolar cells. This interaction results in the presentation of SP-D’s collagenous tails in an aggregated state to calreticulin/CD91 on alveolar cells. The binding of SP-D to this complex facilitates the production of pro-inflammatory cytokines, which are vital for orchestrating the immune response against pathogens. This process not only enhances phagocytosis—where immune cells engulf and digest pathogens—but also initiates broader pro-inflammatory responses that amplify the overall immune reaction (14, 64, 65). These dual interactions of SP-D with SIRP-α and the CD91–calreticulin complex play crucial roles in regulating immune responses in the lung. By modulating these cellular functions, SP-D maintains a delicate balance between inflammatory and anti-inflammatory responses, contributing to overall immune homeostasis in the lung (14, 65).

Initially identified as a protein interacting with the CRD of SP-D, glycoprotein 340 (gp340) was thought to be a receptor for SP-D due to its presence on the surface of alveolar macrophages. Further investigation revealed that gp340 is the same protein as salivary agglutinin, a component of saliva that binds *Streptococcus mutans*, a bacterium associated with dental caries (18, 83). Despite its association with SP-D and its location on cell surfaces, gp340 lacks a transmembrane domain, leaving its role as an SP-D receptor uncertain.

Recent studies have reported that SP-D binds to Toll-like receptors (TLRs), which are family of conserved cellular receptors recognizing pathogen-associated molecular patterns (PAMP) from various sources, including CpG-containing DNA from bacteria, peptidoglycan from Gram-positive bacteria, LPS from Gram-negative bacteria, viral RNA, and yeast. Activation of TLRs initiates inflammatory responses, mediated by cytokines like TNF and IL-1β (8, 18, 52, 64). TLRs recognize PAMPs and damage-associated molecular patterns (DAMPs), initiating immune responses by activating signalling pathways that produce pro-inflammatory cytokines activating critical pathways like NF-κB. By emphasizing the importance of these mechanisms among species, the progressive stability of TLRs and their interaction with SP-D provides knowledge concerning immunity and highlights potential treatment targets for infectious diseases (8, 52, 64, 84).

Some novel discoveries have identified specific receptors for SP-D on monocytes, underscoring its dual role in modulating their functions through the collagen domain. SP-D binds to Leukocyte-associated Ig-like receptor-1 (LAIR1), expressed on neutrophils and monocytes (65, 80). This binding inhibits Fc receptor-mediated reactive oxygen species (ROS) production in human myeloid leukemia cells, enhancing immunomodulatory functions of SP-D. By preventing excessive ROS production, SP-D plays a crucial role in preventing tissue damage during immune responses. The interaction between SP-D and LAIR1 leads to signaling that dampens immune cell activities such as maturation, proliferation, and degranulation, thereby modulating immune activation (80).

Additionally, OSCAR, which is expressed on the surface of interstitial lung and blood CCR2^+^ inflammatory monocytes, binds specifically to the collagenous region of SP-D. This interaction was confirmed using OSCAR-Fc fusion protein, which can capture both recombinant and native SP-D from human bronchoalveolar lavage fluid. When SP-D binds to OSCAR on monocytes, it stimulates the release of TNF-α, demonstrating another mechanism by which SP-D modulates immune responses. This comprehensive understanding of SP-D interactions with LAIR1 and OSCAR underscores its significant role in regulating immune response (65, 72).

SP-D, in some cases of infection (such as Helminth infection), interacts with immune cells, particularly alveolar macrophages, promoting their polarization to an alternatively activated phenotype. This polarization is crucial for effective immune responses and enhances the macrophages’ ability to clear infections. Additionally, SP-D can enhance the production of type 2 cytokines, such as IL-4 and IL-13, which signal through their respective receptors (IL-4Rα) and promote the induction of innate lymphoid cells (ILC2s). These ILC2s are essential for orchestrating the overall immune response (65, 85)

Beyond its immune functions, SP-D also has putative interactions with non-immune receptors that may influence various physiological processes. SP-D is believed to play a role in lipid metabolism and homeostasis within the lungs. It may interact with receptors involved in lipid uptake and processing, contributing to the maintenance of pulmonary surfactant levels, which are critical for reducing surface tension at the alveolar air-liquid interface. Furthermore, SP-D’s interactions are not limited to the respiratory system; it is suggested that SP-D may also have roles in the gastrointestinal tract. SP-D could interact with receptors that regulate gut immunity and the balance of gut microbiota, indicating a broader physiological role that extends beyond respiratory health (8, 84).

## 6 Protection against pathogens by SP-D

SP-D recognizes pathogens through its CRDs, which contains calcium-dependent lectin-binding sites that specifically recognize and bind to certain sugar moieties on the surface of pathogens, including bacteria, viruses, and fungi marking them for clearance by immune cells. The CRD has a high affinity for mannose, N-acetylglucosamine, and fucose, commonly found on the surface of pathogens. It acts as a pattern recognition molecule, identifying specific PAMPs on the surface of pathogens, which allows for the discrimination between self and non-self-molecules (6, 86)

Apart from carbohydrates, SP-D can also bind to lipid and specific proteins component on the surface of certain pathogens, such as *Mycoplasma pneumoniae*, It binds to specific cell wall molecules of fungi, such as 1,3-β-D-glucan, 1,6-β-D-glucan, Galactosaminogalactan, Galactomannan, Glucuronoxylomannan, Mannoprotein 1, and glycosylated proteins facilitating the immune recognition and clearance of fungal infections by host cells. Studies have shown that SP-D binding to fungal hyphae is inhibited in the absence of specific cell wall components, indicating the importance of these interactions in pathogen recognition (14, 87). Further, SP-D has been shown to bind to melanin on the dormant conidia of *Aspergillus fumigatus*. This interaction was not inhibited by sugars, suggesting a different mode of recognition involving protein components on the surface of the fungal pathogens (14, 87).

The binding of HNPs to SP-D has been shown to inhibit SP-D’s hemagglutination-inhibiting activity against Influenza A Virus (IAV), thereby reducing SP-D’s overall antiviral efficacy (67, 88; 40, 65). BALF containing HNPs were less antiviral mainly because the HNPs precipitated SP-D out of the BALF (67, 88). This reduces the ability of SP-D to control replication and prevent IAV-induced inflammatory responses (65, 67, 88). Even though HNPs have antiviral properties of their own, this is usually outweighed by competitive action between HNPs and SP-D in the context of co-treatment leading to lessened overall inhibition because most IAV strains are highly sensitive towards SP-D (65). But still, in some cases they may show cooperative effects against SP-D-resistant strains (67, 88). Furthermore, SP-D specific binding to the sulfated N-acetyl galactosamine moiety of decorin, a small leucine-rich proteoglycan structurally related to collagen type I and secreted by fibroblasts, would also play an important role in immune regulation (40, 69) contributing significantly in pulmonary defense.

SP-D inhibits the ability of viruses like influenza A virus (89), adenovirus, respiratory syncytial virus (RSV) (90, 91), and herpes simplex virus type 1 to enter host cells, leading to aggregation and inactivation of the virion. It binds to bacterial pathogens through interactions with components such as LPS on Gram-negative bacteria and peptidoglycan and lipoteichoic acid on Gram-positive bacteria; also it binds to fungi and yeasts such as *Saccharomyces cerevisiae* and *Candida albicans* via surface glycoproteins (92). By binding to these entities in bacteria and fungi, SP-D can facilitate the aggregation of pathogens, inhibiting their growth and also their clearance through various mechanisms, including opsonization and enhancement of phagocytosis by alveolar macrophages (93).

SP-D binds to a number of bacterial and fungal pathogens (extensively reviewed in the field) and enhances the phagocytic activity of immune cells, such as macrophages and neutrophils, triggering the signalling pathways leading to the activation of immune responses, including the production of proinflammatory cytokines and chemokines that recruit and activate other immune cells (86).

Abnormal levels of SP-D have been associated with hypersensitivity lung diseases, indicating its importance in maintaining immune homeostasis and regulating the inflammatory environment in the lungs. SP-D helps to balance the immune response, preventing excessive inflammation while effectively combating pathogens.

SP-D can disrupt the membrane integrity of certain pathogens, compromising the pathogens’ ability to survive and replicate within the host (94). Additionally, it can directly interact with lipid components of the pathogen’s membrane, leading to membrane disruption and subsequent killing of the pathogen (95). By destabilizing the membrane structure of pathogens, SP-D contributes to the overall defence against microbial infections, ultimately aiding in the recognition, neutralization, and elimination of pathogens to maintain pulmonary homeostasis and protect against respiratory infections.

## 7 Protection against allergens

An allergic reaction is initiated when lung encounter allergens, leading to the release of histamine and other chemicals by basophils. SP-D is released upon allergen exposure, playing an important role in enhancing the immune response against allergens by modulating cellular immune responses and influencing various allergic parameters (18). Allergens come into contact with both pulmonary surfactant and phagocytes in the terminal airways (96). Studies have revealed that SP-D deficiency can impact allergic immune responses, affecting specific IgE binding to allergens and the release of histamine triggered by allergens such as *Aspergillus fumigatus* (*Afu*) (97) and house dust mite (98).

*In vivo* investigations using SP-D-deficient mice have provided insights into the importance of SP-D in modulating the early stages of allergic inflammation (65). These studies suggest that SP-D is essential for regulating the immune response to allergens by influencing cytokine production and the proliferation of lymphocytes (99). Administering SP-D therapeutically resulted in a significant decrease in specific IgE and IgG1 levels, as well as reductions in peripheral blood eosinophilia and pulmonary infiltration, particularly in a murine model of allergic bronchopulmonary aspergillosis (ABPA) using BALB/c mice (100).

Additionally, SP-D and recombinant human SP-D have demonstrated protective effects against allergens such as house dust mite extract (*Dermatophagoides pteronyssinus*) (9, 101) and 3-week culture filtrate (3wcf) of Aspergillus fumigatus (Afu) (9, 86, 100–102).

*In vivo* studies involving treatment of mouse models of ABPA induced by Afu 3wcf with SP-D or rfhSP-D resulted in decreased eosinophilia and lower levels of specific IgG and IgE antibodies, along with decreased levels of IL-2, IL-4, and IL-5, and an increase in IFN-γ in the cultured spleen cells, indicating a significant shift from a pathogenic Th2 to a protective Th1 polarization of the helper T-cell immune response (100). Comparable protective effects have been observed in mouse models of lung allergy caused by *Dermatophagoides pteronyssinus* allergens (9, 103, 104).

SP-D has the ability to bind to starch granules found in grass pollen allergens and promote the engulfment of these granules by alveolar macrophages (105). Additionally, SP-D decreases the growth of peripheral blood mononuclear cells (PBMCs) obtained from children with allergic asthma (106) and suppresses IL-2 production by PBMCs (107) and IL-8 by activated eosinophils (108).

**Figure 2:**
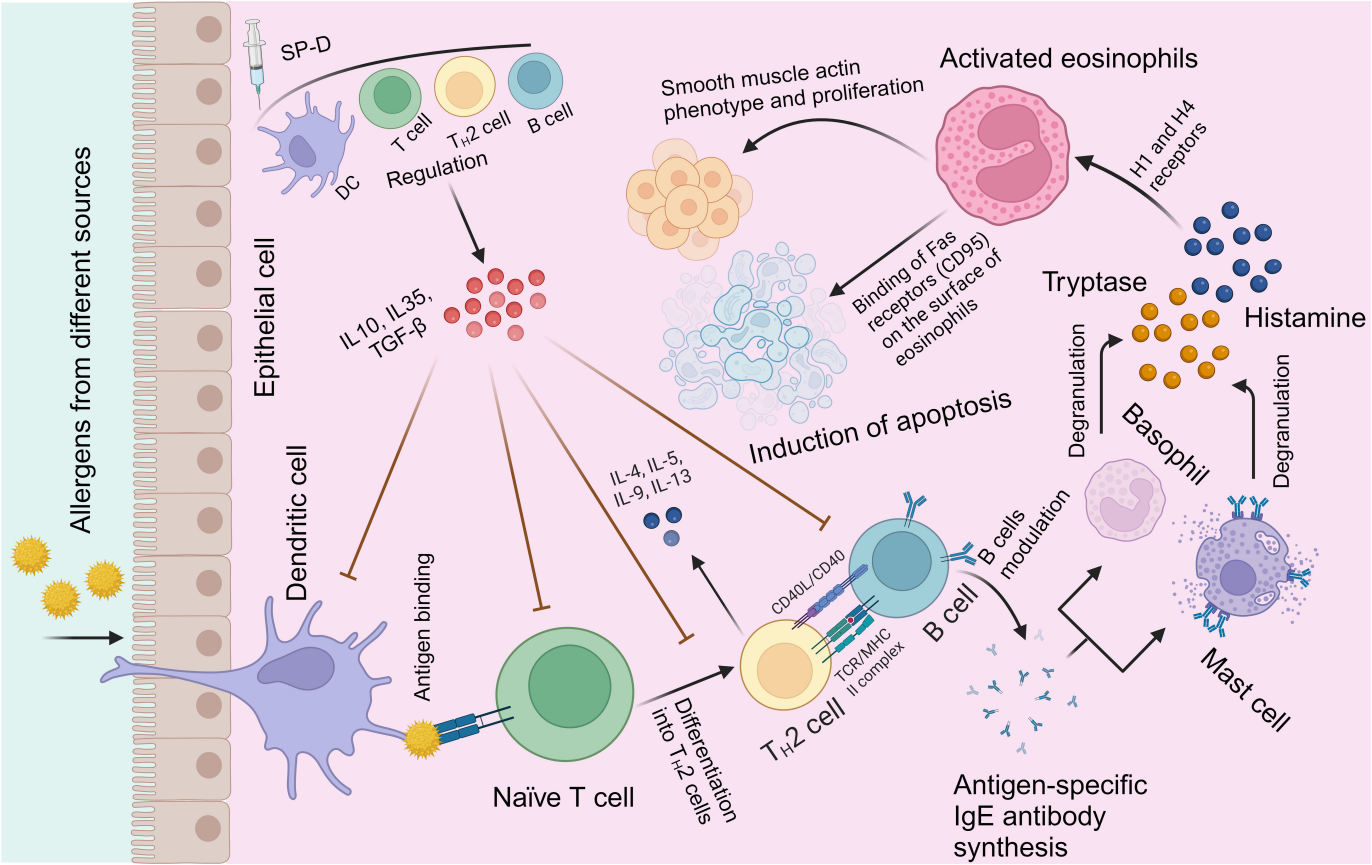
Immunological mechanisms of allergic modulation by SP-D. Exposure to allergens on epithelium initiates a chronic IgE-mediated allergic inflammation facilitated by Th2 cells and B cells (109). SP-D (or its recombinant fragment) stimulates the regulation of allergen-mediated induction of Th2 cells and shifts to a predominantly Th1 response (97, 110). Other protective mechanisms include direct binding by SP-D to allergens; inhibition of allergen binding to specific IgE, of IgE cross-linking by allergen, of IgE-allergen complex presentation, of IgE synthesis by B cells. A clear effect is suppression of histamine release by basophils and mast cells, in addition to induction of apoptosis in activated eosinophils.

SP-D plays a crucial role in lung host defence, not only by regulating the function of innate immune cells but also by interacting with antigen-presenting cells and T cells (15, 65). **Figure 2** illustrates the immunological mechanisms of allergen immunotherapy, which aligns with SP-D role in modulating allergic responses. Recent studies on non-infectious lung diseases and lung injuries suggest that SP-D levels in bronchoalveolar lavage fluid and serum can fluctuate and potentially serve as biomarkers for disease or injury (10, 65). Additionally, the polymorphisms of surfactant proteins (SPs) may lead to altered functions and affect susceptibility to or severity of lung diseases through different receptor interactions (18, 54).

There is a body of evidence to suggest that SP-D is a potent and versatile link between innate and adaptive immunity. SP-D can directly inhibit T cell proliferation by binding to T cells, while SP-A can indirectly inhibit T-cell proliferation by suppressing dendritic cell (DC) maturation (19, 107). Additionally, SP-D has been shown to enhance antigen uptake and presentation (65). Hence, SP-D appears to provide protection against allergenic challenge through multiple mechanisms, including allergen masking, inhibition of allergen-IgE interaction and histamine release, suppression of sensitized basophils, mast cells, or eosinophils activation, as well as modulation of B and T-cell proliferation, dendritic cells (DCs), and macrophages, ultimately leading to Th cell polarization (18, 54, 65, 107). At the B cell level, SP-D has been shown to suppress IgE synthesis by primed B cells ex vivo derived from allergic patients sensitised to grass pollen allergens (111).

SP-D has been shown to inhibit exotoxin-triggered chemotaxis and eosinophil cationic protein degranulation in healthy eosinophils, indicating a regulatory role in modulating eosinophil functions. Moreover, SP-D selectively induces apoptosis in activated eosinophils from allergic asthmatics, highlighting its potential in controlling immune responses (112, 113). Additionally, SP-D exhibits anti-inflammatory properties, regulating immune responses by modulating pathways such as NF-κB to control the production of pro-inflammatory cytokines and mitigate inflammation. In addition to its role in immunity, SP-D is involved in regulating apoptosis, influencing cell survival and clearance processes crucial for tissue homeostasis in the lung and kidney. By aiding in tissue repair and protection through the clearance of apoptotic cells and debris, SP-D supports organ function and integrity, particularly during injury or infection. Moreover, SP-D may mediate lung-kidney cross-talk by influencing immune responses and inflammatory pathways in both organs, underscoring its significance in overall immune defense and organ function (114).

### 7.1 Insights from SP-D knockout mice in innate immunity

The development of SP-D gene knockout mice has provided significant insights into its roles in lung homeostatic and innate immunity. Studies with SP-D knockout mice have demonstrated impairments in immune responses, highlighting the essential role of SP-D in host defence mechanisms and immune surveillance (115). These mice also exhibit increased susceptibility to infections, emphasizing the protective function of SP-D in combating pathogens (89–91, 116). Furthermore, the creation of high-level expressing SP-D transgenic mice offers a promising avenue to further explore the protective role of SP-D in infection resistance. Overall, research using SP-D knockout and transgenic models has deepened our understanding of how SP-D contributes to immune defence, infection susceptibility, and overall host defence mechanisms in innate immunity (117).

The study investigating the effects of truncated recombinant human SP-D on emphysema in SP-D deficient mice revealed the attenuation of lung damage and inflammation. Despite lacking a proper collagenous domain, the truncated rfhSP-D was able to maintain lung structure and create an anti-inflammatory environment, leading to a reduction in the severity of emphysema in the mice. This anti-inflammatory effect was attributed to the binding of the CRD of rfhSP-D to the signal inhibitory regulatory protein α (SIRP α), resulting in the inhibition of NFκB signaling, immune cell activation, and MMP-expression. The study’s results also highlighted the importance of the expression system for the biological activity of the fragment, as rfhSP-D expressed in yeast was not as effective in preventing emphysema development in SP-D knock-out mice compared to E. coli-expressed rfhSP-D. Furthermore, treatment with rfhSP-D led to beneficial effects on lung morphology, including a reduction in emphysema severity, normalization of type II cell numbers, and improvements in the intracellular surfactant pool. These findings suggest that rfhSP-D has the potential to serve as a therapeutic agent for respiratory diseases characterized by decreased levels of SP-D and associated lung damage, offering promise for future treatment strategies in conditions such as chronic obstructive pulmonary disease (COPD) and cystic fibrosis (118).

## 8 Therapeutic effects of surfactant protein-D in lung hypersensitivity

In a murine model of lung hypersensitivity to *Afu* allergens, therapeutic effects of SP-D were observed. The study demonstrated that intranasal treatment with SP-D or rhSP-D (a recombinant fragment of human SP-D containing trimeric C-type lectin domains) significantly lowered eosinophilia and specific antibody levels in mice with Afu-induced pulmonary hypersensitivity. Lung sections of the treated mice showed reduced infiltration of lymphocytes and eosinophils compared to untreated mice (9, 97). Furthermore, the levels of proinflammatory cytokines IL-2, IL-4, and IL-5 were decreased, while the level of the anti-inflammatory cytokine IFN-γ was increased in the supernatants of cultured spleen cells, indicating a shift towards a Th1 response. These findings highlight the therapeutic potential of SP-D in mitigating allergic immune responses and modulating cytokine profiles in a murine model of lung hypersensitivity to *Afu* allergens.

When challenged with *Afu* allergens, mice genetically deficient in SP-D exhibit increased susceptibility compared to wild-type mice. Studies have shown that SP-D gene-deficient mice are more susceptible to pulmonary hypersensitivity induced by *Afu* allergens, displaying intrinsic hypereosinophilia and elevated levels of IL-5 and IL-13, along with a Th2 bias in the immune response. Intranasal treatment with SP-D or recombinant SP-D (rfhSP-D) effectively rescued *Afu*-sensitized SP-D knock-out mice, leading to a significant decrease in IL-13 and IL-5 levels, reduced pulmonary eosinophilia, and improved lung tissue integrity. These findings underscore the critical role of SP-D in offering resistance to pulmonary allergenic challenges and highlight the increased susceptibility of SP-D gene-deficient mice compared to wild-type mice when exposed to *Afu* allergens.

## 9 Protection against cancer

SP-D has been extensively studied for its immune surveillance in cancer progression and prognosis. It has been shown to provide protection against cancer through various mechanisms, including inducing apoptosis, suppressing cell proliferation, and inhibiting cancer progression. It has been found to induce apoptosis in various cancer cell lines and primary cancer cells, including lung, pancreatic, prostate, ovarian, and others (23, 113, 119). Studies have demonstrated SP-D’s selective apoptotic effect on cancer cells with elevated High-Mobility Group A1 (HMGA1) expression, an oncogenic transcription factor overexpressed in various high-grade malignancies, while sparing healthy cells. This suggests its potential as a targeted therapy in cancer treatment, playing a pivotal role in promoting cancer cell survival and proliferation. Inhibiting HMGA1 expression with SP-D disrupts oncogenic signalling pathways that support cancer cell survival, ultimately leading to apoptosis and inhibition of cancer cell growth. This specific molecular interaction highlights the precision with which SP-D exerts its anti-leukemic effects, offering a targeted approach to leukemia treatment that holds promise for the development of effective therapeutic strategies in combating cancers such as acute myeloid leukemia (AML), acute lymphoid leukemia (ALL), and Burkitt’s lymphoma (112, 113, 119).

**Figure 3:**
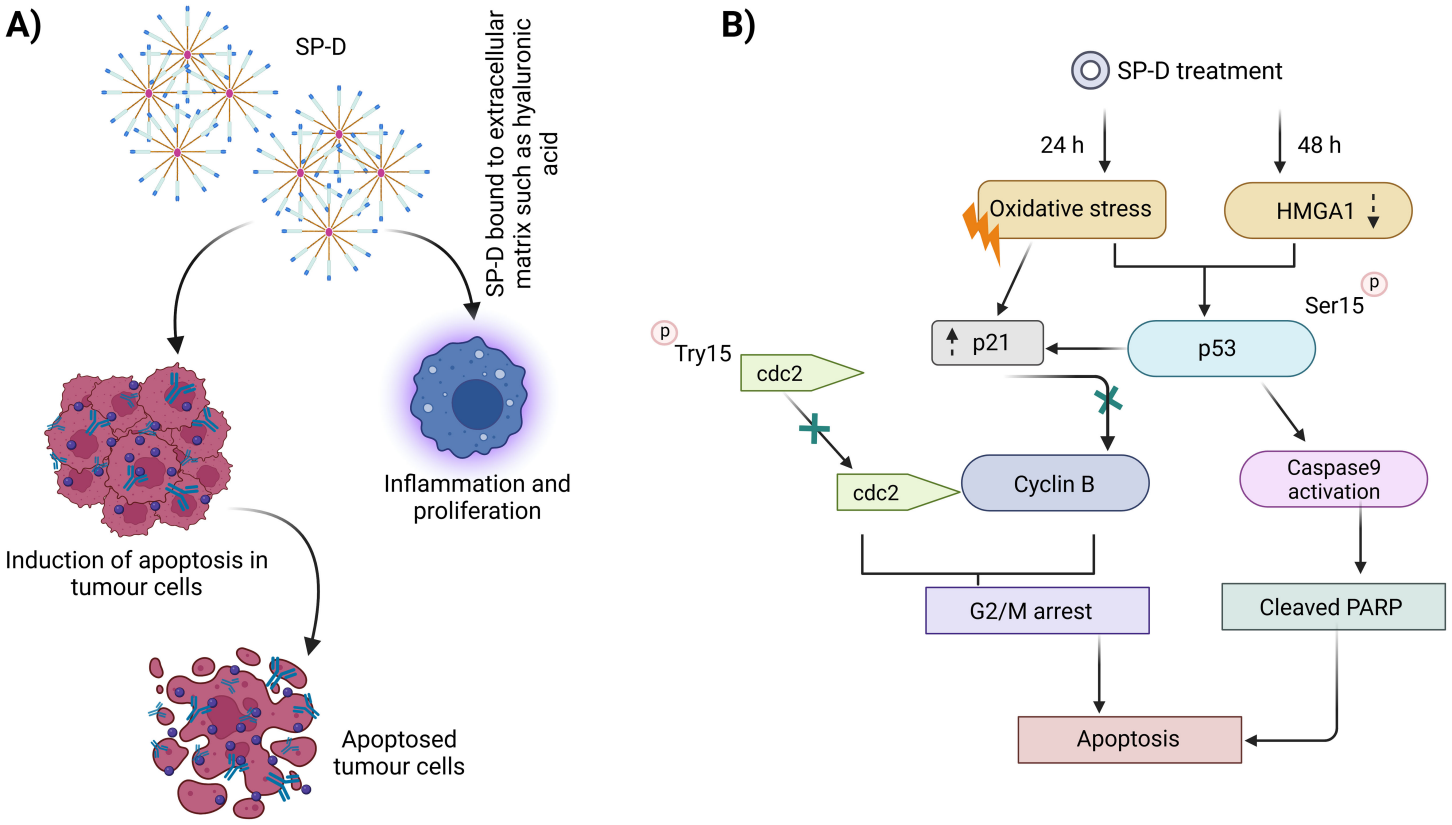
Mechanism of antitumorigenic activity by SP-D or rfhSP-D. Conversely, SP-D may also contribute to a pro-tumorigenic environment **(A)**, enabling tumor growth via interaction with ECM such as hyaluronic acid. A number of stages in the innate immune surveillance against cancer are affected by SP-D, including induction of apoptosis via intrinsic as well as extrinsic mechanisms, suppression of epithelial-to-mesenchymal transition, and potentiation of adaptive immunity **(B)**.

**Figure 3** illustrates the mechanism of antitumorigenic activity, highlighting the role of SP-D in targeting and eliminating oncogenic cells as well as their potential hindrance to a pro-tumorigenic environment (112, 113).

**Figure 4:**
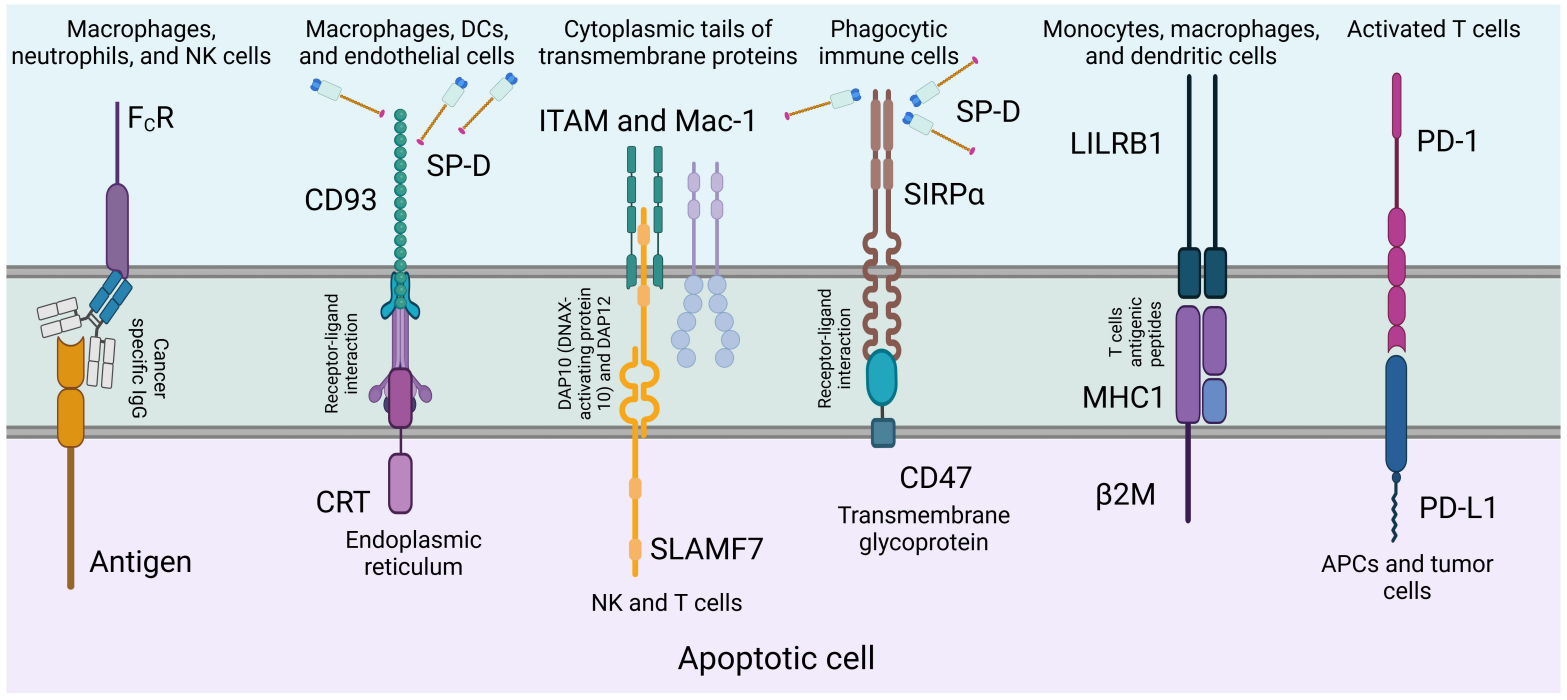
Regulation of phagocytosis initiation signals. Tumor-specific IgG antibodies opsonize cancer cells by binding to tumor antigens. Phagocytic cells, expressing FcR receptors, recognize the particular region of these antibodies, triggering the initiation of antibody-dependent cell phagocytosis (120). SP-D can act as an adapter molecule for the clearance of dying or dead cells that need to be removed. Of a number of known receptors, its association with calreticulin-CD91 complex is noteworthy.

SP-D has been associated with a lower risk of lung cancer development and a better prognosis, as it suppresses cancer progression by downregulating the epidermal growth factor signalling pathway (45). Additionally, SP-D has been found to inhibit cell proliferation, invasion, and metastasis in lung cancer cell lines (23, 45). (**Figure 4**) depicts the regulation of phagocytosis initiation signals, emphasizing how tumor-specific IgG antibodies opsonize cancer cells, which are then recognized by phagocytic cells via FcR receptors, triggering antibody-dependent cell phagocytosis.

In ovarian cancer, SP-D has been shown to be overexpressed in various subtypes, and its expression is associated with poor prognosis (119). Treatment of ovarian cancer cell lines with a recombinant fragment of human SP-D (rfhSP-D) led to decreased cell motility, inhibition of the mTOR pathway activity, and increased caspase 3 cleavage and pro-apoptotic genes Fas and TNF-α (22, 113, 119).

SP-D has a significant effect on pancreatic cancer cells, affecting their morphology, invasion ability, and metastasis. SP-D has been shown to induce apoptosis *in vitro* via the Fas-mediated pathway and inhibit TGF-β expression, thereby reducing invasive potential (22, 45, 113, 119); and it also induces cell cycle arrest in the G1 phase, limiting pancreatic cancer cell proliferation and inhibiting tumor growth. Apart from the apoptosis pathway, rfhSP-D has also shown the ability to interfere with epithelial-to-mesenchymal (EMT) transition, thereby regulating cancer progression and metastasis (113).

However, the role of SP-D in cancer is complex, and its function can be influenced by the tumor microenvironment (22, 119). Further studies are required to delineate the relationship between SP-D, its ligands in the tumor microenvironment, and SP-D receptors on primary cancer cells (23, 45, 113, 119).

## 10 Role in pregnancy

SP-D plays a multifaceted role in pregnancy, contributing to neonatal immunity, pathogen defence, immunoprotection, foetal lung development, amniotic fluid regulation and tissue remodeling during parturition (6, 10, 15, 121, 122). SP-D has been found in the female genital tract, including the vagina, cervix, uterus, fallopian tubes, and ovaries, suggesting its involvement in protecting these organs from infections (15, 121, 123, 124). Its primary functions during pregnancy involve supporting neonatal immunity, regulating inflammation, and providing immunoprotection to both the mother and the developing fetus. SP-D regulates immune responses, protecting the female reproductive tract from infections and ensuring surfactant balance in the fetal lungs by blocking pro-inflammatory cytokines. This maintains immune equilibrium and prevents harmful inflammation for both the mother and fetus (15, 124). The role of SP-D in pregnancy extends to neonatal immunity, where it helps regulate immune responses in newborns and protect them from infections. Studies have shown that SP-D deficiency in offspring can lead to increased susceptibility to pathogens, highlighting the importance of SP-D in enhancing neonatal immunity and immune defences (15). SP-D also plays a role in tissue remodeling during parturition and potentially controlling inflammation during pregnancy. SP-D interacts with decorin, a proteoglycan found in fetal membranes and the uterine cervix, in human amniotic fluid. The concentration of SP-D and decorin is inversely related, suggesting a regulatory role in intrauterine tissue remodeling during labor (6, 121). This interaction may facilitate cervical ripening and dilatation during labor and regulate the tensile strength of foetal membranes at the end of pregnancy (6, 123). SP-D levels in amniotic fluid rise with gestational age, although less pronounced compared to SP-A, leading to a change in the SP-A/SP-D ratio. SP-D is involved in recognizing and clearing pathogens from fetal membranes and amniotic fluid, potentially reducing the risk of intrauterine infections and protecting against conditions like chorioamnionitis, which can lead to preterm birth (6). The levels of SP-D in the amniotic fluid are good biomarkers of fetal lung maturation (6, 121, 123). Furthermore, SP-D has been found to modulate the prostaglandin pathway, which is crucial for maintaining pregnancy and regulating the onset of labor (122).

## 11 SP-D polymorphisms in human health and disease

SP-D is a calcium-dependent defense lectin located on mucosal surfaces and also present in bronchoalveolar lavage, serum, and amniotic fluid (8, 15). The synthesis of SP-D is typically influenced by genetic factors (single nucleotide polymorphisms), alterations in structural proteins, and environmental conditions, making them a therapeutic target in pulmonary disorders (125). SP-D gene consists of 8 exons and its polymorphisms are seen on 3’UTR (untranslated region), protein coding region and 5’UTR that influence SP-D concentration mainly in serum as well as lungs, activity and disease associations (126, 127). Genetic mutations of the SP-D gene in human and mice disrupt the surfactant homeostasis and pulmonary innate immunity (128, 129).

Human SP-D is composed of trimeric subunits and multimeric assemblies of these subunits, stabilized by N-terminal interchain disulfide crosslinking. SP-D is produced by alveolar type II pneumocytes in a range of oligomeric forms: trimers (the smallest protein unit secreted by these cells), hexamers, dodecamers, and larger oligomers that differ in size, referred to as “fuzzy balls” (130–132). Dodecamers and higher-order oligomers are considered the most active oligomeric forms of the protein, particularly for C-type lectin-mediated activities. Multimeric SP-D organisation endows anti-microbial and anti-inflammatory characteristics, whereas trimeric subunits are proposed to exacerbate inflammation (133).

The major SP-D single nucleotide polymorphisms (SNPs) are A538G (rs2243639) and C92T (rs721917). rs2243639 polymorphism correlates with an increased risk of chronic obstructive pulmonary disease (COPD) in Caucasian populations, whereas the rs721917 polymorphism shows a similar association in Asian populations (134). Previous research has demonstrated that Rs721917 is linked to heightened vulnerability to tuberculosis (135, 136), Influenza A virus infection, allergic rhinitis (137, 138), COPD (139), and respiratory syncytial virus (RSV) infection in infants (140), atopy, and elevated blood levels of SP-D (141). Conversely, rs2243639 has been linked to ulcerative colitis and susceptibility to RSV in neonates (142, 143).

SP-D gene polymorphism, rs721917, is associated with Gestational Diabetes Mellitus (GDM), suggesting a regulatory role of SP-D in GDM (144). The SP-D polymorphism, rs721917 C/T, is associated with a greater susceptibility to acute kidney injury development in the Chinese population. Increased plasma SP-D level has been shown to be associated with a higher risk of the development of acute kidney injury in patients with sepsis (145).

SP-D is present in female reproductive system and is known to protect against intrauterine infections (146). SP-D polymorphisms (rs1923534 in 5’UTR, rs721917 in +32 T>C, Met11Thr, rs2243639 in +478 G>A, Ala160Thr, and rs3088308 in +868 T>A, Ser270Thr) are associated with respiratory outcome in preterm babies. Polymorphisms rs1923534, rs721917 and rs3088308 are associated with higher risk of respiratory distress, need for oxygen supplementation and respiratory support (147). However, various factors that influence neonatal health include SP-D genetic polymorphisms, gestational age, method of delivery, and maternal/environmental microflora (148).

The serum distribution of low molecular weight (LMW) trimeric units of SP-D and high molecular weight (HMW) species is dependent upon an SNP variant in the SP-D gene, specifically rs721917 (149). The prevalence of LMW SP-D in axial spondyloarthritis may be regarded as a unique molecular signature that could amplify local or systemic inflammatory responses (150). An N-terminal structural polymorphism (Met11Thr) and its accompanying O-glycosylation have been previously demonstrated to correlate with inadequate multimerization and a relatively low proportion of multimeric Thr11 SP-D compared to Met11 SP-D (149). Multimerization has demonstrated significance in enhancing microbial phagocytosis.

## 12 Plant-based expression and production of human SP-D

The property of SP-D to recognize specific sugar residues, similar to how plant lectin’s function, can be used to trigger immune-like responses in plants. For example, SP-D could bind to pathogenic carbohydrate structures in plants, initiating a defence response and providing a mechanism for disease resistance. Moreover, SP-D might enhance stress tolerance by stabilizing cell membranes, regulating stress response pathways, and increasing the expression of stress-related proteins. This approach can provide a valuable tool for developing crops tolerant to environmental challenges. Further research could lead to producing SP-D in plants for potential therapeutic approaches.

There has been some research considering the potential for expressing mammalian SP-D in plants for biotechnological purposes (151). For example, molecular farming might be used to express SP-D in plants to overcome enhanced pathogen resistance or to produce SP-D for pharmaceutical uses. Molecular farming involves using genetically engineered plants and plant cell suspension cultures as platforms for the large-scale, cost-effective production of recombinant pharmaceutical proteins (152). This involves inserting the gene that encodes SP-D into the plant genome, allowing the plant to produce the protein. Compared to mammalian and bacterial systems, plants have several advantages for producing recombinant SP-D proteins (153). They have significantly lower production costs due to no requirement of expensive growth media or complex processes, unlike mammalian, bacterial, or insect bioreactors (154). Compared to the traditional systems, the upstream production in plants only requires light, water, and basic nutrients for growth.

Plants can perform complex post-translational modifications, which are crucial for the biological activity of many mammalian proteins (155). Targeting proteins to specific intracellular compartments in plants can influence post-translational modifications and improve yields. Chloroplast transformation with SP-D could be considered as much effective with high level of expression and avoiding gene silencing. In addition, plants pose a much lower risk of introducing human or animal pathogens during downstream processing, a concern with mammalian cell-based production (156). Moreover, the eukaryotic protein synthesis pathway is well conserved between plants and animals, enabling plants to potentially fold and assemble complex multimeric proteins like SP-D (157). This method can be scaled up on an agricultural level, overcoming the need for costly bioreactors and reducing the dependence on highly skilled labor, making it an attractive option for industries (158). One of the most commonly used plant-based expression platforms is tobacco, which has a high biomass yield and is neither a food nor a feed crop, minimizing the risk of transgenic material entering the food chain.

However, despite these advantages, downstream processing in plant systems remains a major challenge, as it is more expensive compared to mammalian or microbial systems (159, 160). This stage of production, which involves the extraction and purification of the protein, can account for up to 80% of the total production costs of therapeutic proteins (161). This high cost is due to the complexity of the purification process, which must effectively remove plant-specific contaminants and ensure the protein’s safety and efficacy.

Bustos et al. (162) focused on the production, purification, and characterization of three recombinant human surfactant protein D (rhSP-D) variants in tobacco plants, involving full-size hSP-D, its truncated form hNCRD, and an hNCRD-DsRed fusion protein. The variants were expressed in both the apoplast and cytosol. Transgenic tobacco plants with apoplast-targeted expression produced higher yields of rhSP-D compared to mammalian and bacterial systems (162). However, purification had challenges due to strong binding of rhSP-D variants to chromatography resin and plant-derived contaminants, leading to low yields post-purification. The enzyme linked immunosorbent assay (ELISA) showed the lectin-binding activity of most variants, though those with C-terminal His6 tags failed to bind ligands. Despite only partial purification, the untagged full-size hSP-D and hNCRD-DsRed fusion protein demonstrated bacterial agglutination activity, at lower levels than a control protein and with activity loss after two months of storage (162).

Therefore, it is found that further research is needed to develop efficient purification methods that retain the biological activity of plant-derived rhSP-D. These advancements in genetic engineering and plant biotechnology will reduce the downstream costs and will enhance the yield and quality of SP-D, making plant-based production systems more competitive with traditional methods.

## 13 Future work and perspectives (clinical trials, plant-based drug delivery, etc)

Understanding the intricate role of surfactant protein D (SP-D) in immune responses holds promising therapeutic implications across various clinical scenarios. Its multifaceted role in pathogen recognition, immune modulation, and inflammation regulation holds immense promise for novel therapeutic interventions across a spectrum of respiratory conditions. Firstly, this knowledge could catalyse the development of innovative therapies targeting SP-D or its associated pathways, potentially revolutionizing treatments for respiratory infections, inflammatory lung diseases, cancers and other conditions where SP-D plays a pivotal role. Manipulating SP-D levels or activity could bolster host defence mechanisms against pathogens, offering a potential avenue to strengthen immune responses in individuals with compromised immunity or other ailments.

In the realm of therapeutic development, understanding SP-D’s intricate mechanisms paves the way for innovative treatment strategies. Recombinant forms of SP-D or specific fragments of the protein emerge as potential therapeutic agents aimed at bolstering innate immune responses, clearing infections, and curbing inflammation in respiratory conditions. These therapeutic approaches hold promise in combating antibiotic-resistant pathogens and preventing secondary infections, addressing a pressing concern in global healthcare.

Moreover, modulating SP-D levels or activity presents a novel avenue for immune modulation in respiratory diseases characterized by immune dysregulation, such as asthma, chronic obstructive pulmonary disease (COPD), and acute respiratory distress syndrome (ARDS). By restoring immune balance, SP-D-targeted therapies offer a beacon of hope for alleviating inflammation and improving clinical outcomes in these conditions. Personalized medicine approaches based on individual SP-D profiles and immune response characteristics could also be envisioned, tailoring treatments to optimize outcomes for patients.

The antimicrobial properties of SP-D also offer a potential breakthrough in controlling infections. Strategies aimed at enhancing SP-D’s antimicrobial functions or utilizing recombinant SP-D as a therapeutic agent hold promise in combating a myriad of pathogens.

In the pursuit of therapeutic excellence, the development of innovative drug delivery systems holds immense potential. Plant-based drug delivery platforms emerge as a promising avenue for the targeted delivery of SP-D-based therapies to the lungs. Leveraging the biocompatibility and versatility of plant-derived carriers, such as nanoparticles or liposomes, enables precise delivery of therapeutic agents to the respiratory mucosa, optimizing treatment efficacy while minimizing systemic side effects.
